# Association of poly-purine/poly-pyrimidine sequences with meiotic recombination hot spots

**DOI:** 10.1186/1471-2164-7-179

**Published:** 2006-07-18

**Authors:** Andrew TM Bagshaw, Joel PW Pitt, Neil J Gemmell

**Affiliations:** 1 School of Biological Sciences, University of Canterbury, New Zealand; 2 Bioprotection and Ecology Division, Lincoln University, New Zealand

## Abstract

**Background:**

Meiotic recombination events have been found to concentrate in 1–2.5 kilo base regions, but these recombination hot spots do not share a consensus sequence and why they occur at specific sites is not fully understood. Some previous evidence suggests that poly-purine/poly-pyrimidine (poly-pu/py) tracts (PPTs), a class of sequence with distinctive biochemical properties, could be involved in recombination, but no general association of PPTs with meiotic recombination hot spots has previously been reported.

**Results:**

We used computational methods to investigate in detail the relationship between PPTs and hot spots. We show statistical associations of PPT frequency with hot spots of meiotic recombination initiating lesions, double-strand breaks, in the genome of the yeast *S. cerevisiae *and with experimentally well characterized human meiotic recombination hot spots. Supporting a possible role of poly-pu/py-rich sequences in hot spot recombination, we also found that all three single nucleotide polymorphisms previously shown to be associated with human hot spot activity changes occur within sequence contexts of 14 bp or longer that are 85% or more poly-pu/py and at least 70% G/C. These polymorphisms are all close to the hot spot mid points. Comparing the sequences of experimentally characterized human hot spots with the orthologous regions of the chimpanzee genome previously shown not to contain hot spots, we found that in all five cases in which comparisons for the hot spot central regions are possible with publicly available sequence data, there are differences near the human hot spot mid points within sequences 14 bp or longer consisting of more than 80% poly-pu/py and at least 50% G/C.

**Conclusion:**

Our results, along with previous evidence for the unique biochemical properties and recombination-stimulating potential of poly-pu/py-rich sequences, suggest that the possible functional involvement of this type of sequence in meiotic recombination hot spots deserves further experimental exploration.

## Background

Crossovers between chromosomes occur during meiotic cell division resulting in heritable genetic recombination. These crossovers have a complex, non-random distribution, and in the last decade recombination hot spots 1–2.5 kilo bases (kb) wide have been experimentally well characterized in yeast, mice and humans (reviewed in [1,2]). Hot spots show a wide range of crossover frequencies, which are occasionally several hundred times greater than the level expected if crossovers were distributed randomly across chromosomes. Experimental studies that have located multiple hot spots in contiguous regions of the human genome have found that they often occur in clusters, which are separated by regions of about 50–100 kb showing very low recombination frequencies [3-5].

Hot spots do not share a consensus sequence, and the mechanisms responsible for regulating their distribution and activity levels are not well understood, but several molecular features of hot spot recombination have been described (reviewed in [6]). These include a locally open chromatin structure, presumably allowing access to recombination machinery [7,8], and a requirement for a chromosomal double-strand break (DSB) to initiate recombination [9,10]. A less well explained feature of hot spots is the influence on activity levels of sequence context, including flanking sequences several kb away [11-13]. This suggests that epigenetic, or distal sequence, factors may have a greater influence than local sequences on hot spot regulation, and consistent with this idea are recent studies showing that the locations of hot spots in humans and chimpanzees do not correspond despite more than 98% sequence similarity between the two species [14,15].

Local sequences clearly have some influence on hot spot recombination, however, as activity levels can be sensitive to local, single nucleotide changes [16-19]. The molecular basis of this sensitivity is largely unexplained, with the exception of the *Schizosaccharomyces pombe *a*de *6 hot spot, in which a single nucleotide change promotes transcription factor-binding, creating the hot spot [20,21], though its activity is also influenced by flanking sequences [22,23]. Other sequences have been found to be associated with hot spots including GC-rich DNA [24], tandem repeats [19,25], transcription factor binding sites [26-28], poly-A/T tracts [29] and some specific motifs less than 10 bp long [30]. Direct experimental evidence for a role of such sequence features in hot spot activity is lacking in most cases, the exceptions being transcription factor binding sites [20,21,26-28], and a 14 bp poly-A/T tract, deletion of which was found to reduce gene conversion activity at the yeast *ARG4 *hot spot by 75% [29].

Human recombination rates estimated across windows hundreds of thousands to millions of bp wide have been reported to correlate negatively with poly-A/T tract frequencies [31], but positive correlations with broad scale recombination rates have been shown for other simple sequences, including the class of sequence with purines (A or G) on one strand of the DNA duplex, and pyrimidines (T or C) on the complementary strand, of which poly-A/T is a subset [31,32]. Experimental evidence also suggests that poly-purine/poly-pyrimidine (poly-pu/py) tracts (PPTs) in general deserve further attention, for example they have been shown to affect recombination [33], replication [34], and gene expression [35] in model systems and these effects have often been linked to the ability of PPTs with some GC-content readily to form stable intramolecular secondary structures under physiological conditions [33,34,36,37]. These structures can be sensitive to single nucleotide changes [38,39], but cannot be formed by poly-A/T [40], though poly-A/T duplex DNA can form intermolecular three-stranded aggregates with synthetic oligonucleotides [41]. Recently, a study predicting hot spot locations throughout the human genome based on statistical analysis of haplotype data found several poly-pu/py-rich motifs of 5–9 bp to be associated with hot spots [30], providing further support for the possible importance of poly-pu/py-rich sequences in meiotic recombination.

We investigated in detail the relationship between PPTs and hot spots in humans and the yeast *S. cerevisiae*. We show significantly elevated PPT frequencies in hot spots in both species. We also found that the three single nucleotide changes shown to be associated with human hot spot recombination rates all occur in high GC-content poly-pu/py-rich sequences of at least 14 bp, and that sequence differences between humans and chimpanzees in regions where there is a hot spot in humans but none in chimpanzees occur in similar poly-pu/py-rich sequence contexts.

## Results and discussion

### Hot spots investigated

Recent experimental studies in humans [3-5] and yeast [24] have reported multiple meiotic recombination hot spots and cold regions across contiguous segments of genomic DNA, allowing comparison of sequence patterns between the two types of regions. In the yeast *S. cerevisiae*, recombination intensity has been assayed indirectly throughout the entire genome using microarray analysis of DSB frequency patterns [24]. This study identified 303 hot and 49 cold open reading frames (ORFs), and, combining adjacent ones, defined 177 hot spots, which encompassed all previously known hot spots in the species, and 40 cold spots. For the purposes of our investigation, we extended the hot and cold spots to include the intergenic regions (IGRs) adjacent to the ORFs concerned, since yeast hot spots are typically centred on IGRs, in which most DSBs occur [42]. The hot spots as we defined them had a mean length of 3466 bp. DSBs have also been mapped to 76 much narrower sites on *S. cerevisiae *chromosome 3 [42], but many of these had very low levels of DSBs and may therefore indicate background recombination events rather than hot spots as normally defined, so we analysed them separately.

In humans, multiple hot spots have been mapped by experimental methods in the MHC Class II region on chromosome 6, in which seven hot spots have been identified over 292 kb [4,5,43], and in a 206 kb segment of chromosome 1, in which eight hot spots have been mapped [3,19]. In each region, areas between hot spots showed very low levels of recombination. Two other human hot spots have been well characterized experimentally. In the Beta-Globin gene cluster, a hot spot was mapped to within 11 kb, with a 90 kb adjacent cold region also identified [44]. The location of the Beta-Globin hot spot was later narrowed to a region of under 2 kb [45]. Finally, in the pseudoautosomal region of the Y chromosome, a 9.9 kb section of the *SHOX *gene was assayed for recombination and found to contain a hot spot [46]. All of the human hot spots included in our analysis were 2.5 kb or less in size. We limited our investigation in humans to these 17 well-characterized hot spots known at the time of writing. Hot spots have also been predicted throughout the human genome using statistical analysis of haplotype data to infer past recombination events [30], but recent evidence indicates that these methods are not always reliable for predicting hot spots in the present generation [3,5,47,48].

### Hot spots show elevated frequencies of PPTs

We used a pattern-matching computer algorithm to detect PPTs in the hot spots and cold regions reported in the above-mentioned studies. A 12 bp PPT has been shown to form a stable intramolecular quadruplex [49], but in our search of the literature we did not find reports of shorter PPTs forming stable secondary structures, so we initially searched for PPTs of at least 12 bp. In yeast, mean frequencies per kb were 1.92 in hot spots, and 0.97 in cold spots, which was a statistically significant difference (p = 1.74 × 10^-10^). Because most DSBs occur in IGRs [42], we repeated the analysis excluding ORFs and found that the difference between hot spots and cold spots increased, with the mean per kb frequencies 3.93 in hot spot IGRs and 1.62 in cold spot IGRs (p = 1.65 × 10^-9^). The mean length of PPTs of at least 12 bp was 15.49 bp in hot spots and 14.22 bp cold spots, also a statistically significant difference (p = 0.0036). In yeast, there were no significant differences between hot and cold spots for mean PPT GC-content.

**Figure 1:**
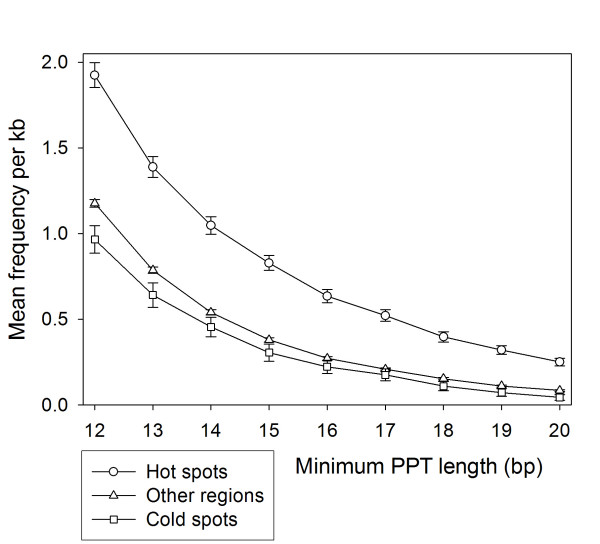
**Association of high PPT frequencies with yeast hot spots**. Mean frequencies of PPTs with length minima between 12 and 20 bp in 177 hot spots of meiotic double-strand breaks (DSBs) in the genome of the yeast *S. cerevisiae *compared with the mean frequencies in 40 DSB cold spots and 233 other regions comprising the remainder of the genome sequence. Error bars are plus and minus one standard error of the mean.

Transcription factors can bind to PPTs as short as 5 bp [50], and we wished to know whether very long PPTs were associated with hot spots, so we varied the minimum PPT length between 5 and 40 bp. We found greater mean PPT frequencies in hot spots than cold spots, with the differences statistically significant, for length minima 7 through 33. Frequencies for the 233 remaining regions of the genome not classified as hot or cold spots had similar frequencies to cold spots, showing that the differences were primarily due to elevated frequencies in hot spots rather than lowered frequencies in cold spots (Figure 1).

The 17 human hot spots did not show significantly elevated PPT frequencies for length minima 5–12 bp. Increasing the length minimum increased the ratio of frequencies between hot spots and cold regions, however, with the differences statistically significant for minima of 13 bp (means per kb 2.02 in hot spots and 1.33 kb in cold regions, p = 0.045), 14 bp (means per kb 1.50 in hot spots and 0.99 in cold regions, p = 0.036), and 15 bp (means per kb 1.21 in hot spots and 0.73 in cold regions, p = 0.036). In humans, no significant differences between hot spots and cold regions were found for mean PPT length, but human hot spot-associated PPTs had a higher mean GC-content (45.4%) than those in cold regions (37.5%), which was a statistically significant difference (p = 0.001). The difference was not due to a high GC-content ratio between hot spots and cold areas (1.00). Evidence indicates that high GC-content PPTs are more likely to form secondary structures [40,51-53], so we repeated the searches looking only for PPTs with GC-contents above the mean, as calculated across all PPTs of at least 12 bp found in the study regions (38% G/C). We found that this limitation increased the levels of enrichment of PPTs in human hot spots. The associations were statistically significant for size minima 13 bp (mean frequencies per kb 1.48 in hot spots and 0.795 in cold regions, p = 0.0079) and 14 bp (mean frequencies per kb 1.04 in hot spots and 0.533 in cold regions, p = 0.017). In contrast, the association between PPTs and hot spots in yeast was weaker for high GC-content PPTs.

Because PPTs with mismatches to the homopurine/homopyrimidine motif are much more common than pure PPTs, we repeated all the searches allowing some mismatches. In humans, mean frequency differences for tracts of at least 20 bp (means per kb 1.51 in hot spots and 0.80 in cold regions, p = 0.022) and 24 bp (means per kb 0.92 in hot spots and 0.34 in cold regions p = 0.036) were statistically significant for PPTs with one mismatch per 10 bp allowed. No significant differences were found with one mismatch allowed every 5 bp. In yeast, allowing mismatches generally reduced the frequency ratios between hot spots and cold spots, but did increase the greatest size minimum for which the difference was statistically significant from 33 bp (for pure PPTs) to 39 bp when one mismatch was allowed per 10 bp, and 41 bp when one mismatch was allowed per 5 bp.

In both yeast and humans hot spot intensities vary greatly, so we asked if hot spot intensity was correlated with PPT frequency. We found no significant correlations in humans, but in yeast we did find significant positive correlations between previously reported hot spot intensity [24] and mean PPT frequencies with the length minima 18, 23, 25 and 26 bp, and for minima 26, 29 and 32 through 34 with one mismatch allowed per 10 bp. Even longer PPTs were correlated when one mismatch was allowed per 5 bp, with significance for minima 33, 35 and 37 through 39. The correlations were weak in all cases, however, with coefficients ranging between 0.15 and 0.19 (Spearman's rho).

The lack of a strong correlation between PPT frequency and hot spot intensity suggests that a high density of PPTs is not in itself a primary determinant of hot spot activity levels. High PPT density is also not sufficient in itself to cause a hot spot, since hot spot locations in humans and chimpanzees do not correspond despite more than 98% sequence similarity between the two species [14,15]. High PPT frequency could be a factor in hot spots, however, and it is also possible that only certain types of PPT may be involved in recombination. Below, we discuss evidence that PPTs could stimulate recombination through secondary structure formation and/or protein binding. Exact sequence requirements are not fully understood, either for binding of recombination-related proteins, or secondary structure formation by PPTs. It is possible, therefore, that the association between PPT frequency and hot spots may be due to a greater likelihood that regions with a high frequency of PPTs will contain a functional tract.

### PPTs are associated with DSB sites mapped with a resolution of about 500 bp

Baudat and Nicolas (1997) mapped meiotic DSBs throughout chromosome 3 of the genome of the yeast *S. cerevisiae *and identified 6 ORFs and 70 IGRs subject to at least one DSB [42]. Overall, these DSB-containing ORFs and IGRs averaged 567 bp in length. We found that 53 of the 70 DSB-containing IGRs had at least one PPT of 12 bp or longer (76%), and 35 of them had a PPT of at least 15 bp (50%). Of the 92 IGRs on the chromosome without a mapped DSB, 56 had a PPT of at least 12 bp (61%), and 26 had one of at least 15 bp (28%). Frequencies of PPTs with length minima 5–15 bp were significantly elevated in DSB-containing IGRs compared with the remaining IGRs on the chromosome. The strongest enrichment was observed with a 15 bp minimum length (p = 0.000791); mean per kb frequencies 1.83 in DSB-containing IGRs and 0.925 in IGRs without a DSB. When mismatches were allowed, mean frequencies were greater in DSB-containing IGRs than non-DSB IGRs with the differences statistically significant for size minima 10 through 23 bp with one mismatch allowed per 10 bp, and 11 through 26 bp with one mismatch allowed per 5 bp. We found no significant differences between the 6 DSB-containing ORFs and other ORFs on the chromosome.

Most of the 70 DSB-containing IGRs showed very low levels of DSBs (see Figure 2 in ref [42]), and 48 of them occurred outside hot spots reported in the genome-wide survey by Gerton and co-workers [24]. It is therefore likely that many of them indicate non-hot spot background recombination events, since these have been found to occur with low frequency between hot spots [3-5]. For PPTs of at least 15 bp, the mean frequency per kb was 1.70 in DSB-containing IGRs outside hot spots reported by Gerton *et al*. [24]. This was significantly greater than the mean per kb frequency of 0.925 found in IGRs without a DSB (p = 0.00262).

### PPTs are associated with broad hot spot-containing regions

Because PPTs have been shown to stimulate formation of recombination intermediates at distances as great as 4000 bp [54], we wished to know if they were associated with broad hot spot-containing regions. In yeast, we compared frequencies of PPTs of at least 12 bp between IGRs and ORFs flanking hot spots, and IGRs and ORFs within cold spots. Comparisons were made up to a maximum distance of 4 IGRs from hot spots. IGRs one ORF removed from hot spots and IGRs two ORFs removed from hot spots showed significantly higher PPT frequencies than cold spot IGRs, indicating a regional association between PPT frequency and hot spot-containing areas. The mean PPT frequency of 2.90 per kb in the PPT-enriched hot spot-flanking IGRs was significantly less, however, than the mean per kb frequency of 3.93 in hot spot IGRs. The mean distance encompassed by the hot spot-containing regions in which PPTs were enriched was just over 11.5 kb. We found no significant differences between ORFs flanking hot spots and cold ORFs.

**Figure 2:**
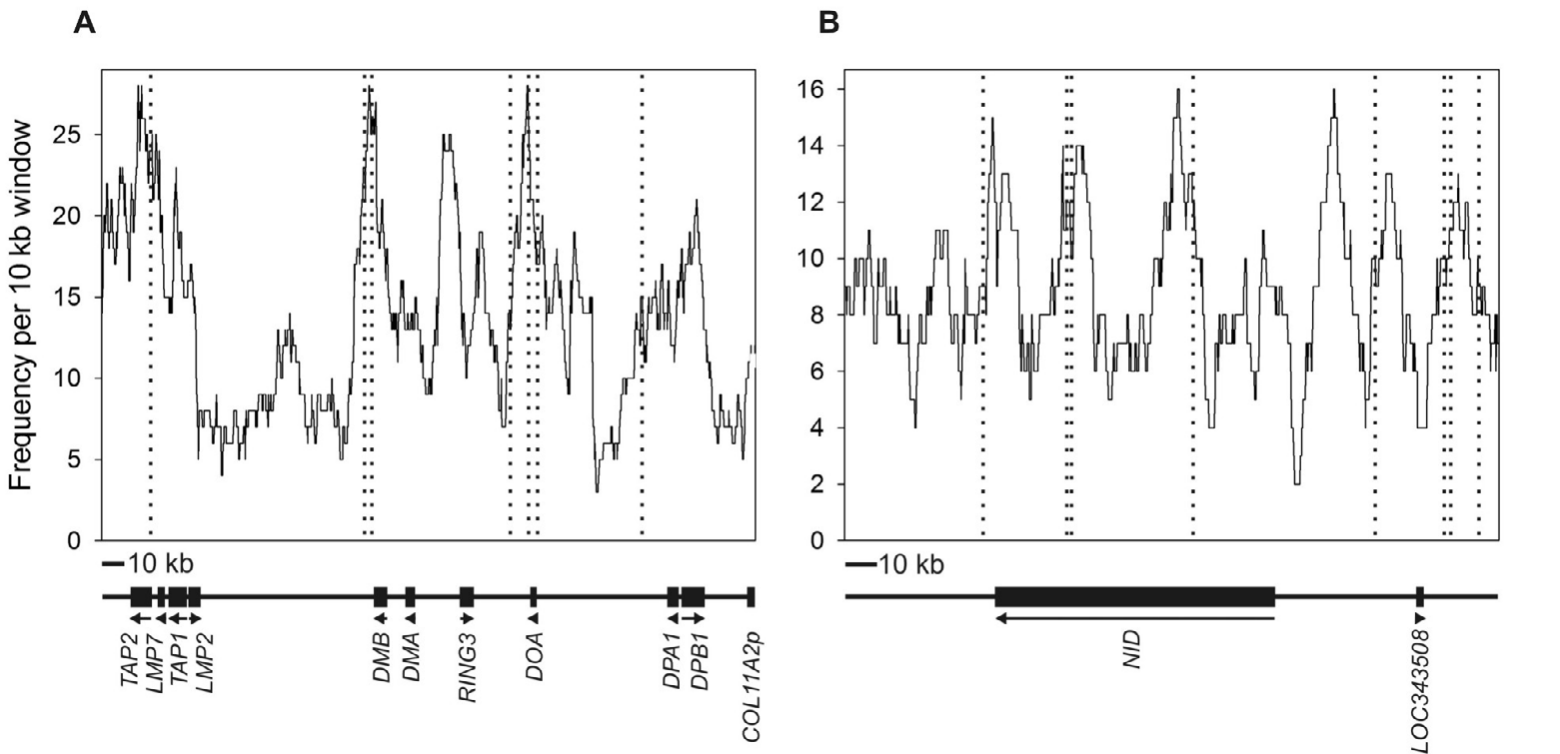
**Densities of high GC-content PPTs relative to human hot spot locations**. Sliding window plots of the densities of PPTs of at least 12 bp, with GC-contents above the mean for PPTs over the regions we investigated, relative to hot spot locations in A): a 292 kb region of the human MHC Class II region in which 7 hot spots are known and B): a 206 kb region of human chromosome 1 in which 8 hot spots are known. Vertical dotted lines represent hot spot mid point locations. Sliding windows were 10 kb wide and moved in steps of 100 bp. Locations of genes in the regions are shown below the plots with arrows indicating direction of transcription.

In humans, we investigated PPT frequencies for all 17 hot spots in windows of increasing size centred on hot spot mid points, which we will refer to as hot regions. PPT frequencies in hot regions 3 to 40 kb wide were compared with remaining cold regions, which were defined as experimentally mapped cold regions lying outside these windows. We found no significant differences for PPTs of at least 12 bp, except when low GC-content tracts were excluded. PPTs of at least 12 bp with greater than the mean PPT GC-content had consistently higher frequencies in hot regions than remaining cold regions with the differences statistically significant for window sizes 3, 4, 9, 10, 12, 14 through 22, and 24 kb. Excluding the hot spot sequences themselves from the analysis weakened the associations, but they remained significant for window sizes 4, 10, and 15 through 20 kb. Sliding window plots of the densities of high GC-content PPTs in the two regions in which multiple human hot spots have been mapped showed that peaks in density often occur within a few kb of hot spots. For PPTs of at least 12 bp, this was most striking with window sizes of about 10 kb (Figure 2). No significant regional associations were found for high GC-content PPTs in yeast.

### The associations are not primarily due to microsatellite PPTs

One cause of the associations we observed might be a mutation bias resulting from recombination, or other properties of hot spot regions, acting to cause expansion of PPTs. With regard to this possibility, the degree to which the hot spot-associated PPTs consist of short, direct tandem repeats (STRs, or microsatellites) is relevant, because microsatellites have well-described mutational dynamics [55], but it is unclear how a mutation bias could act on non-repetitive PPTs. We therefore asked whether hot spots contained an elevated proportion of microsatellite PPTs. Using a separate search for PPTs of at least 12 bp consisting of short tandem repeats with a repeated unit 6 bp or less (PP-STRs), we found mean per kb frequencies in yeast of 0.41 in hot spots and 0.13 in cold spots. Poly-A/T made up a large proportion of these, with mean per kb frequencies of 0.27 in hot spots and 0.059 in cold spots. After subtracting the number of PP-STRs of at least 12 bp from the number of PPTs of all kinds of at least 12 bp for each hot spot and cold spot, we found that the difference in mean per kb frequencies was still significant (1.51 in hot spots and 0.84 in cold spots, p = 1.19 × 10^-6^). In humans, PP-STRs of at least 12 bp were more frequent in cold regions than hot spots. This was also the case for poly-A/T considered separately. While not ruling out the possibility of a mutation bias, these results suggest that any bias that may be operating to cause an excess of PPTs in hot spot regions is probably not primarily due to insertions causing duplications of adjacent sequence, which is how microsatellites expand [55].

### Sequence polymorphisms suggesting a function in hot spots for high GC-content poly-pu/py-rich sequences

At the time of writing, there are three known cases in humans of single nucleotide changes associated with altered recombination levels in hot spots [16,18,19]. All three polymorphisms were associated with several-fold reductions in recombination frequency and were located close to the estimated hot spot mid points. We found that each of these polymorphisms occurs within 3 bp of the end of a sequence 14 bp or longer consisting of 85% or more poly-pu/py and at least 70% G/C (Table 1).

Known features of hot spot recombination are similar across very diverse taxa (reviewed in [6]), so comparison of PPTs between humans and chimpanzees in the regions of human hot spots where there is no hot spot in chimpanzees might provide interesting data. To date, there are six cases in which the recombination activity of chimpanzee chromosomal regions orthologous to experimentally characterized human hot spots has been investigated, and in all these cases little or no evidence for hot spot activity was found in chimpanzees [15]. Because the human hot spot activity-associated polymorphisms all occurred within 166 bp of the hot spot mid points [16,18,19], we aligned the human and chimpanzee sequences 166 bp on either side of the human hot spot mid points. We found that all five of the hot spot central regions for which alignments are possible contain differences within sequences 14 bp or longer consisting of more than 80% poly-pu/py and at least 50% G/C (Table 2). In three out of the six sequences, there were differences within 3 bp of their ends, and four out of six contained a difference in the terminal 4 bp. The analysis did not include the *DNA2 *hot spot, because the publicly available chimpanzee sequence was missing 206 of the 332 bp region centred on the hot spot mid point, including the mid point itself. The *DMB2 *hot spot was included in the analysis, but the chimpanzee sequence was missing 34 bp from the central region. Within these limitations, 11 out of the total of 43 differences within 166 bp of the human hot spot mid points occurred within high GC-content, poly-pu/py-rich sequences as defined above. Over all the central regions we found 9 such sequences, 6 of which contained at least one difference between humans and chimpanzees. Of the 33 sequences of this type located within hot spots but outside the central regions, 10 contained differences. The differences were probably not due to recombination or other features of hot spots preferentially causing mutations in this type of sequence, because similarity between the two species in poly-pu/py-rich sequences as defined was 96.73% in hot spots, which was only slightly lower than the 97.27% overall sequence similarity for the 5 hot spots.

The observation that all three human hot spot recombination frequency-associated polymorphisms known at the time of writing occur in poly-pu/py-rich high GC-content sequences suggests that certain sequences of this type may be involved in determining hot spot activity. This is somewhat supported by the similar sequence contexts found for the human/chimpanzee differences. The particular PPTs that we have identified as possibly having functional roles in hot spots (Tables 1 and 2) might prove to be useful targets for experimental studies. Secondary structure formation and protein binding by the sequences are consistent with the data, and could be investigated further.

Although the sequence requirements for poly-pu/py-rich sequences to form secondary structures are not fully understood [52,53,56], the structures can tolerate a substantial proportion of interruptions to the homopurine/homopyrimidine motif [34,36,53], and can also be sensitive to single nucleotide changes [38,39]. They have been shown to stimulate inter-plasmid recombination [33] and dimerization [57], as well as recombination hot spot activity in *E. coli *[58]. Immunocytological evidence shows that PPTs do form secondary structures on human chromosomes *in vivo *[59], but the possibility that this occurs in meiotic recombination hot spots has not to our knowledge been tested.

The additional, non-exclusive, possibility that certain poly-pu/py-rich sequences could contain binding motifs for proteins that act to stimulate recombination is supported by evidence that PPTs can bind transcription factors [50,60], since transcription factor binding is a determining factor in some yeast hot spots [20,21,26-28]. Binding of the nuclear matrix-associated type III intermediate filament proteins is also a possibility. Intramolecular quadruplex secondary structures, which can be formed by poly-pu/py-rich, GC-rich sequences [53], have been shown preferentially to bind these proteins *in vitro *[61].

### Speculation on possible mechanisms by which PPTs could influence hot spot recombination

There are several possible mechanisms by which PPTs could act as functional components in recombination hot spots. It is conceivable that PPTs could mediate recombination by being themselves the sites of DSB formation, possibly as a result of nuclease action on secondary structures. This seems unlikely to be a common mechanism, however, since DSBs are found at many positions over 100–500 bp regions in yeast hot spots, and are probably position-specific rather than sequence-specific (reviewed in [2]). PPTs may also function in hot spots by altering local chromatin structure. This could result from their previously demonstrated involvement in the formation of DNase I hypersensitive sites, which are nucleosome-free regions of chromatin [62,63]. An open chromatin structure is one important factor in hot spots, presumably allowing access to the recombination machinery [7,8]. Binding of transcription factor proteins may help to achieve this in at least some hot spots, since opening up of chromatin structure is one function of transcription factors, and this has been proposed as the most likely reason for the requirement shown by some yeast hot spots for transcription factor binding (reviewed in [2]). The possible role of PPTs in hot spots may also be mediated by transcription factors, since PPTs in promoter regions can affect transcription, and evidence suggests that this is due to opening of the chromatin structure by the PPT *via *secondary structure formation and/or binding of transcription factor proteins (reviewed in [60,64]). Not all promoter regions are hot spots, however, so factors other than an open chromatin structure are clearly involved.

The potential of PPTs to cause replication pausing (reviewed in [65]) may also be relevant to their possible function in hot spots. A presently unexplained spatial and temporal coupling between DNA replication and meiotic recombination has been demonstrated in yeast [66], and it has been suggested that replication pausing could promote DSB formation *via *localized modification of histones [2]. Another property of PPTs suggesting a mechanism by which they could promote recombination is their ability to stick together, forming multi-stranded aggregates [41,67]. This suggests the possibility that they may help homologous chromosomes to align prior to meiotic recombination, and it has been proposed that this could be mediated by Hoogsteen base pairing interactions [67].

## Conclusion

Our results, along with previous evidence for the unique biochemical properties and recombination-stimulating potential of poly-pu/py-rich sequences, suggest that the possible functional involvement of this type of sequence in meiotic recombination hot spots deserves further experimental exploration. Relevant tests include deleting PPTs from hot spot regions in model organisms and assaying the effects on recombination. Another possible approach would be to test the structure-forming and protein-binding capabilities of hot spot-associated PPTs *in vitro*.

## Methods

We used pattern-matching algorithms programmed in C to search for PPTs and PP-STRs in genomic DNA sequence. Where possible, we used the sequence versions used in the studies that reported the hot spot locations Yeast sequences and ORF locations were downloaded from the Stanford website [68]. The GenBank accession numbers for the 16 yeast chromosomes are NC_001133 through NC_001148. The GenBank accession numbers for the human hot spot sequences are: Beta-Globin hot spot: GI:37541814, chromosome 1 hot spots: GI:37549514, and *SHOX *hot spot: U82668. For the MHC hot spots we used the 28 October 1999 version of the MHC class II region sequence, since that was the version to which the reported hot spot locations corresponded [4]. This version is available at the Sanger Centre website [69]. The GenBank accession numbers for the chimpanzee sequences were as follows: *DNA*3: NW_108387.1, Beta-Globin: NW_113864.1, *DNA*2, *DMB*1 *DMB*2 and *TAP*2: NW_107937.1.

PPTs overlapping hot and cold regions were excluded. When we investigated PPT frequencies in windows with increasing length centred on human hot spots, some of the windows included areas for which recombination rates are unknown, and these were excluded from the analysis. Areas where windows overlapped were combined. When we searched for sequences with mismatches to the poly-pu/py motif we disallowed mismatches in the terminal two bp of tracts. When we searched for sequences at least 80% poly-pu/py rich, we imposed the additional restriction that no 6 bp segment of any sequence was allowed more than two mismatches. Alignments were performed using the BLAST algorithm [70].

Statistical comparison of means (Student's T-test and Mann-Whitney U Test, 2-tailed tests in call cases) and correlation analyses (Spearman's Rank Test) were done using SPSS with significance inferred where p < 0.05 in all cases. All samples were initially tested for normality (Shapiro-Wilk Test) and significantly non-normal samples were subjected only to the non-parametric tests. In cases where yeast hot spots contained multiple ORFs, we used the mean reported ORF rank [24] for the correlation analysis of hot spot intensity and PPT frequency.

## Authors' contributions

ATMB conceived and designed the experiments, analyzed the data and wrote the paper. JPWP wrote the computer programmes. NJG contributed to the interpretation of the data and the writing of the paper.

## Figures and Tables

**Table 1:** Poly-pu/py-rich sequence contexts of polymorphisms associated with hot spot activity in humans. Sequence contexts of the three polymorphisms associated with reduced recombination frequencies in human hot spots. The recombination-suppressing alleles are shown in lower case.

**Hot Spot**	**References**	**Sequence context**	**Distance from hot spot mid point (bp)**
MS32	[19]	(G/c)GTGGGAAGGGTGG	151
*NID*1	[3, 18]	CC(C/t)CCCACCCCACCCC	64
*DNA*2	[4, 16]	AGGGGGCAGCAACAGGG(A/g)GG	166

**Table 2:** Poly-pu/py-rich sequence contexts of differences between humans and chimpanzees in human hot spot central regions. Differences between humans and chimpanzees within 166 bp of human hot spot mid points occurring in sequences at least 14 bp long consisting of over 80% poly-pu/py and at least 50% G/C. In each case, the hot spots are absent in chimpanzees. The chimpanzee sequence is shown in lower case.

**Hot Spot**	**References**	**Sequence context**	**Distance of difference from human hot spot mid point (bp)**
Beta-Globin	[15, 45]	CCTTTTCCCCTCCTACCCCTACTTT(C/t)T	133
*DMB*1	[4, 15]	GGGGGTGAGCAACAG(A/g)AA	35
*DMB*2	[4, 15]	GAGAGGAGAGAAGTGGAGTGGGAGAAAGAAGGAGAG(G/a)GACATAGAGAGAGGGA(G/a)(G/a)GAGGGGAG(C/-)GG(-/a) GGGAAG/AGAGAGAGAGCTGGA	115, 98, 97, 88, 85
*DMB*2	[4, 15]	GGGAGGGGAG(G/t)TGGA	51
*DNA*3	[4, 15]	GGGAGGCC(A/g)AGG(C/a)GGG	23,27
*TAP*2	[4, 15]	(C/g)CTTCATTCCATCC	83
